# Comparative Study of the Effectiveness of sub mucosal Partial Inferior Turbinectomy and Out fracture of Inferior Turbinate in the Nasal Respiratory Function of Rhinoplasty Patients

**DOI:** 10.1007/s00266-019-01423-4

**Published:** 2019-07-01

**Authors:** Mahmood Omranifard, Mehrdad Adib, Sajad Ebrahimpour Boroujeni, Farbod Dadkhah Tirani, Samira Asadi

**Affiliations:** 1 Plastic Surgery Faculty of Medicine Isfahan, Isfahan University of Medical Sciences, Isfahan, Iran; 2 Isfahan University of Medical Sciences, Isfahan, Iran; 3 Barret Honors College, Arizona State University, Tempe, USA; 4 Faculty of Health, Shahrekord University of Medical Sciences, Shahrekord, Iran

**Keywords:** Rhinoplasty, Inferior nasal concha, Respiratory problems, Sub mucosal partial inferior turbinectomy, Out fracture of inferior turbinate, SNOT-22

## Abstract

**Introduction:**

The inferior turbinate is a critical and dynamic structure during rhinoplasty in the internal valve. Many surgeons try to preventively reduce its resistance against the path in the post-rhinoplasty period. To this end, the two methods of “sub mucosal partial inferior turbinectomy” and “inferior turbinate out fracture” are compared in the present study.

**Methods:**

In this clinical study, 110 rhinoplasty candidates were randomly divided into two groups, namely sub mucosal partial inferior turbinectomy and out fracture of the inferior turbinate. To assess the complications, the Sino-Nasal Outcome Test (SNOT-22) was used prior to surgical intervention, and 1, 2, 3, 6, and 12 months following the rhinoplasty procedures.

**Results:**

Based on the results of this clinical study and according to the SNOT-22 questionnaire, there was no significant difference between the two groups prior to surgery and a month following the surgery (*P* > 0.05). However, the average SNOT-22 score for the sub mucosal partial inferior turbinectomy group was significantly lower than that of the group with the out fracture of the inferior turbinate, 2, 3, 6, and 12 months following the surgery.

**Conclusion:**

Both “sub mucosal partial inferior turbinectomy” and “out fracture of inferior turbinate” are effective methods in improving the respiratory function of rhinoplasty patients, yet the former method is more effective than the latter as regards improving the respiratory function of patients.

**Level of Evidence IV:**

This journal requires that authors assign a level of evidence to each article. For a full description of these Evidence-Based Medicine ratings, please refer to the Table of Contents or the online Instructions to Authors www.springer.com/00266.

## Introduction

Rhinoplasty surgery is one of the most common types of aesthetic plastic surgical procedures. Breathing problems are among the most serious complications occurring after rhinoplasty, and entailing the dissatisfaction of patients. In certain studies, 10% of the patients complained about residual or new breathing problems following the primary rhinoplasty. In 70% of the patients undergoing revision rhinoplasty, breathing problems are the main complaints [1]. Among the causes of such breathing problems are blockages due to turbinate hypertrophy, septum deviation, and an overall reduction in the size of the nose and nostrils [2, 3]. Inferior turbinate hypertrophy is one of the complications in rhinoplasty procedures encountered by most plastic surgeons. To prevent iatrogenic injuries, plastic surgeons must master the anatomy and physiology of the nose [4]. Certain inflammatory disorders such as allergic rhinitis or vasomotor lead to inferior turbinate hypertrophy, with ensuing precipitation of collagen in the nasal mucous membrane and hyperplasia, and an increase in the secretion of nasal glands. Septum deviation more often than not co-occurs with inferior turbinate hypertrophy [5]. Surgical interventions treat hypertrophy of inferior turbinate in cases where there is no response to treatment with medications [6]. The inferior turbinate is one of the bones involved in the side wall of the inside of the nose. The inferior turbinate is the largest bone of the nasal cavity and is usually longer in male patients [7, 8]. Turbinate bones are responsible for the steady airflow inside the nose [9]. The inferior turbinate has the most impact on airway resistance and hence is commonly reduced in size so as to improve nasal airflow and increase the space inside the nasal cavity [10]. Sub mucosal partial inferior turbinectomy out fracture is routinely done in rhinoplasty [11]. In sub mucosal partial inferior turbinectomy, the mucous membrane and inferior turbinate bone are resected from the one-third proximal to reduce the surface area of the turbinate. This method enhances the respiratory function in patients and prevents blockage due to hypertrophy of the inferior turbinate bones [12]. Compared with other methods, less damage is involved in “out fracture of inferior turbinate,” where turbinates are broken out and upward relative to the septum [13]. So far, there has not been a similar, accurate, and compendious study to assess patients’ quality of life (QOL) and satisfaction following the rhinoplasty procedure. According to the literature, various methods have been invented to evaluate the outcome of a rhinoplasty such as SNOT and SCHNOS. SNOT-22 could be used in routine clinical practice to underscore the impact of nasal disease in each patient and to measure the outcome and effectiveness of the surgical intervention. The present study aimed to evaluate the nasal function with the assumption that patients have an acceptable appearance. The purpose of this study was to compare the effectiveness of sub mucosal partial inferior turbinectomy and out fracture of inferior turbinate methods concerning the respiratory function of rhinoplasty patients by use of QOL measures and SNOT-22 (Sino-Nasal Outcome Test).

## Methods

In this clinical study, 110 rhinoplasty candidates at *Isfahan University* plastic surgery clinics and private clinics were randomized from 2015 to 2017.

Inclusion criteria were: 1—patients should be interested and consent to rhinoplasty procedure, 2—Armenoid formed nose (Armenoid race), and 3—patients should pass the mental health evaluations.

Exclusion criteria were: 1—significant nasal airway obstruction due to septum deviation and skeletal anatomy, 2—turtose shape septum, 3—breathing problems due to allergies, 4—untreated sinusitis, 5—presence of tumor or nasal polyps, 6—previous rhinoplasty surgery, 7—nasopharyngeal or airway complications, and 8—serious systemic diseases and those with a history of smoking or drug abuse. Our turbinectomy in the two groups was preventive. We tried to match the two groups through clinical examination and CT scan, if needed. The nose was assessed clinically, and because rhinomanometry is influenced by environmental factors, the obtained results are not correct [10].

A complete comprehensive assessment was done on each patient prior to their entry to the clinical study, and photographic and X-ray findings were recorded if needed. Patients who did not follow the appropriate suggested care instructions during the study, or were not cooperative, or experienced new nasal trauma or smoking were omitted from the study [10]. After obtaining the demographic information, such as age, gender, and marital status, patients were randomly divided into two groups using random allocation software. The first group was treated with sub mucosal partial inferior turbinectomy, and the second group was treated with the out fracture of the inferior turbinate method. In this study, patients were divided into two groups: sub mucosal partial inferior turbinectomy, including 10 male and 45 female patients, and out fracture of the inferior turbinate, including 6 male and 49 female patients.

The Sino-Nasal Outcome Test (SNOT-22) questionnaire was the data gathering tool in this study.

This questionnaire is designed to assess the respiratory function of patients and includes 22 questions on the need to blow the nose, nasal blockage, runny nose, sneezing, coughing, postnasal discharge, thick nasal discharge, ear fullness, ear pain, dizziness, facial pain/pressure, reduced sense of smell/taste, difficulty falling sleep, waking up at night, lack of good night’s sleep, waking up tired, fatigue during the day, reduced productivity, reduced concentration, frustrated/restless/irritable states, sadness, and embarrassment in patients. In this questionnaire, each question receives a score from 0 to 5, and hence, the entire questionnaire will have a score ranging from 0 to 110. The higher the overall score of the questionnaire is, the more severe the respiratory disturbances experienced by the patient will be [14]. This questionnaire was completed by each patient prior to surgery and also 1, 2, 3, 6, and 12 months following the surgery. Patients were further evaluated regarding their nasal mucus dryness.

The assessment was performed using the patients’ own statements, as well as endoscopic and frequent speculum examination before and after mucus dryness and cracking or hemorrhagic spots in the nasal cavity; the results were recorded in the checklist, and there were no postoperative complications, although bleeding and dryness are common complications after turbinectomy.

All patients kept the tampon in the nose in the postoperative period as in other rhinoplasty methods. Mucosal dryness was observed in the early months after surgery, which was gradually reduced and improved by saline drops, spray, and lubricant.

## Implementation Method

Before dissecting the nose, the nasal cavity was opened with a speculum. In the inferior turbinate out fracture method, a fracture was produced at the turbinate and nasal wall junction, and the turbinate was pressed out with the speculum. In the sub mucosal partial inferior method, the mucous membrane was incised at the inferior part, the mucosa was separated from the bone, osteotomy was carried out in the distal part, and the bone was removed from the submucosa.

### Statistical Analysis

To calculate the sample size, the confidence interval was considered to be 99% (equal to a significance level of 2.34), and it was assumed that the sub mucosal partial inferior turbinectomy method has a success rate of 40% and the out fracture of the inferior turbinate has a success rate of 20% (according to the previous studies) [15]; the sample size of 55 for each group was further calculated. Information and data were entered into SPSS Statistics Version 22. To compare the two groups with respect to qualitative data, the Chi-square test was used. To analyze the quantitative data, the independent *t* test and Mann–Whitney *U* test were employed. Further utilized was ANOVA with repeated tests so as to analyze the changes in quantitative data at different time intervals. Quantitative data are represented using standard deviations, and qualitative data are shown through numbers (percentage). *P* values lower than 0.05 were interpreted as a significant correlation.

## Results

There was no significant difference between the two groups with respect to demographic information such as age, gender, and marital status (*P* > 0.05). Demographic data are demonstrated in Table 1.

**Table 1:** Demographic data of two groups of patients

	Out fracture of inferior turbinate	Partial inferior turbinectomy	*P* value
Number	55	55	–
Gender			
Male	(10.9%) 6	(18.2%) 10	0.27^*^
Female	(89.1%) 49	(81.8%) 45	
Age (mean ± standard deviation)	4.95 ± 25.89	4.86 ± 27.05	0.89^**^
Marital status			
Married	17(30.9%)	20(36.4%)	0.54^*^
Single	38 (69.1%)	35 (63.6%)	

*Chi-square test, **Independent t test

The score of the SNOT-22 questionnaire was calculated for the two groups prior to the surgery and 1, 2, 3, 6, and 12 months after. There was no significant difference between the two groups concerning the SNOT-22 questionnaire prior to surgery and 1 month after the surgery (*P* > 0.05). However, the average score of the SNOT-22 questionnaire in the sub mucosal partial inferior turbinectomy group was significantly lower than that of the group receiving out fracture of the inferior turbinate 2, 3, 6, and 12 months following the surgery (*P* < 0.05) (Table 2).

**Table 2:** SNOT-22 scores in different time intervals between two groups

SNOT-22	Out fracture of inferior turbinate	Partial inferior turbinectomy	*P* value^*^
Prior to surgery	12.76 ± 7.79	14.56 ± 7.32	0.13
1 month after surgery	25.61 ± 10.79	23.96 ± 8.54	0.67
2 months after surgery	18.01 ± 7.90	13.43 ± 6.09	0.001
3 months after surgery	13.58 ± 6.08	8.10 ± 3.79	0.0001>
6 months after surgery	10.40 ± 5.80	4.52 ± 3.39	0.0001>
12 months after surgery	7.38 ± 5.28	2.74 ± 3.11	0.0001>

*Mann–Whitney test, SNOT-22: Sino-Nasal Outcome Test

**Fig. 1:**
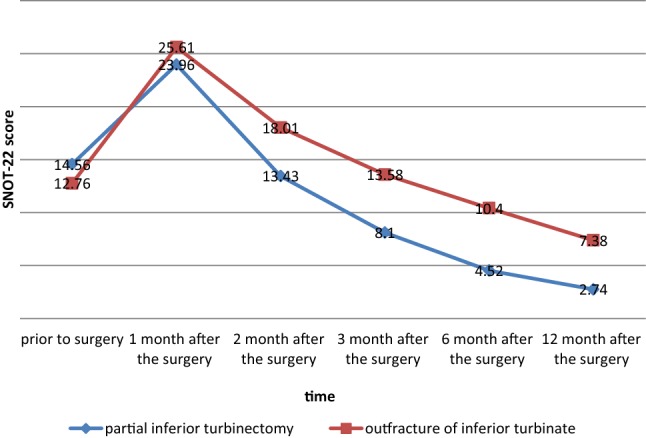
Changes in the SNOT-22 scores in different time intervals. According to the linear diagram, the SNOT-22 scores of both methods were similar up to 1 month, but the SNOT-22 score of the sub mucosal partial inferior turbinectomy group was lower during months 2–12, indicating the better efficacy of this method

According to ANOVA with a repeated test, changes in the SNOT-22 scores were significant at different time intervals (*P* < 0.0001) (Fig. 1).

## Discussion

Based on the results of this study, the SNOT-22 score for the sub mucosal partial inferior turbinectomy group was significantly lower than the score for the out fracture of the inferior turbinate group. Therefore, patients treated with sub mucosal partial inferior turbinectomy experienced less obstruction of the airways and had better overall respiratory functions. Based on the questions asked from the patients, none of the sub mucosal partial inferior turbinectomy patients experienced dryness of the nasal mucus in the long term (3, 6, and 12 months after surgery). In most studies, inferior turbinate reduction is known as a preferred novel method, and sub mucosal partial inferior turbinectomy and out fracture of the inferior turbinate methods are among the most common techniques [16], while our study confirms that the patient had a better feeling and breathing after 1 month. Gandomi studied the effects of partial turbinectomy in rhinoplasty patients, in which one group was treated with both partial turbinectomy and rhinoplasty and one group was only treated with rhinoplasty. It was observed that the respiratory mean scores before and after rhinoplasty in both groups were significantly different. In the partial turbinectomy group, symptoms such as nasal blockage were improved, while other symptoms were not. Further observed was that partial turbinectomy did not cause any significant complications [17]. In a study conducted by de Moura et al. [18], it was concluded that sub mucosal partial inferior turbinectomy is a reliable surgical technique that does not entail any serious complications; this method, however, is not effective in improving the quality of life in short-term periods following the surgery. In the present research, no significant difference was observed concerning the SNOT-22 questionnaire up to 1 month after the surgery. This means that the sub mucosal partial inferior turbinectomy method was not effective in improving patients’ respiratory function in the short term. The out fracture method is a common method that has relatively favorable results in rhinoplasty patients. In this study, all patients had the same postoperative period, and early complications such as bleeding and late complications such as dryness, leading to mucosal ulceration, were not observed in the groups. In a study by Nassif et al. [15], sub mucosal cauterization of the inferior turbinate with and without out fracture was assessed with the conclusion that out fracture has more favorable outcomes. In some studies, it is mentioned that the out fracture method is the optimal approach to reducing the area of the inferior turbinate [19]. In a study done by Buyuklu, 10 patients were treated with septoplasty and out fracture of the inferior turbinate; it was concluded that the latter is an effective and stable method that can be used to improve the nasal airways in mild-to-moderate cases of inferior turbinated hypertrophy with the least amount of complications. With only a few studies done to compare the effectiveness of sub mucosal partial inferior turbinectomy and out fracture of inferior turbinate in rhinoplasty patients, ours is the first to compare the effectiveness of these two methods in improving the respiratory function following rhinoplasty surgery. According to the present research and the aforementioned studies, both sub mucosal partial inferior turbinectomy and out fracture of the inferior turbinate are very effective in improving the respiratory function of patients in the short run (up to 1 month after surgery). On the other hand, in the long run (more than 2 months into the surgery), the sub mucosal partial inferior turbinectomy method was more effective. Moreover, sub mucosal partial inferior turbinectomy does not ensue the dryness of the mucosal membrane in the long term (2, 3, 6, and 12 months after surgery).

### Limitations

The lack of a similar study in the Middle East and the low sample size are the limitations of this study; however, it can pave the way for future studies.

## Conclusion

Both “sub mucosal partial inferior turbinectomy” and “out fracture of the inferior turbinate” are effective methods for improving the respiratory function of rhinoplasty patients, yet the former method is more effective than the latter as regards improving the respiratory function of patients.
